# Elevated expression of growth-regulated oncogene-alpha in tumor and stromal cells predicts unfavorable prognosis in pancreatic cancer

**DOI:** 10.1097/MD.0000000000004328

**Published:** 2016-07-29

**Authors:** Shuijin Lian, Xiaolu Zhai, Xudong Wang, Huijun Zhu, Shu Zhang, Wei Wang, Zhiwei Wang, Jianfei Huang

**Affiliations:** a Department of Oncology; b Department of Laboratory Medicine; c Department of Pathology; d Department of General Surgery, Affiliated Hospital of Nantong University, Nantong, Jiangsu, China.

**Keywords:** Growth-regulated oncogene-alpha, Pancreatic cancer, Prognosis

## Abstract

Growth-regulated oncogene-alpha (GRO-α) has been reported to be over-expressed in a series of human cancers including colorectal cancer, melanoma, gastric cancer, hepatocellular carcinoma, and ovarian cancer and was known to regulate multiple biologic activities associated with tumor progression. But the role in human pancreatic cancer remains unclear. To examine the expression of GRO-α and its clinical significance in pancreatic cancer (PC), a total of 12 fresh PC specimens and 12 surrounding normal tissues to detect *GRO-α* mRNA expression were measured by quantitative real-time polymerase chain reaction (qRT-PCR). Immunohistochemical analysis of GRO-α protein was performed in 160 formalin-fixed, paraffin-embedded PC tissue samples and 68 control specimens, including 37 matched normal surgical margins and 31 benign pancreatic lesions. Kaplan–Meier survival and Cox regression analyses were performed to evaluate the prognosis of PC patients.

Expression of *GRO-α* mRNA in PC tissues was significantly compared with that in adjacent normal tissues (1.399 ± 0.165 vs. 0.870 ± 0.103 *t* = 1.75, *P* = 0.012), GRO-α protein expression in cytoplasm of cancer cells and stroma was detected in 41.88% and 40.63% PC specimens, respectively, and was significantly higher than that in corresponding normal tissues (*P* = 0.008, *P* = 0.002, respectively). High GRO-α expression in the cytoplasm of cancer cells was related to tumor location (*P* = 0.047), tumor status (T classification; *P* = 0.001), distant metastasis (*P* < 0.001), and tumor node metastasis (TNM) stage (*P* < 0.001). High GRO-α expression in the stroma correlated with perineural invasion (*P* = 0.010), T classification (*P* = 0.006) and TNM stage (*P* = 0.004), and was marginally associated with metastasis (*P* = 0.056). Elevated expression of GRO-α in cytoplasm of cancer cells (hazard ratio [HR] = 5.730, *P* = 0.007) and stroma (HR = 3.120, *P* = 0.022) were independent prognostic factors of pancreatic cancer. T classification (HR = 2.130, *P* = 0.023), lymphatic metastasis (HR = 4.211, *P* = 0.009) and TNM classification (HR = 0.481, *P* = 0.031) were also prognostic predictors in PC patients.

GRO-α expression was elevated in pancreatic cancer tissues and might be a potential therapeutic target and prognostic marker in patients with pancreatic cancer.

## 1 Introduction

Pancreatic cancer (PC) ranks the fourth principal reason of cancer-related death in western countries^[1]^ and the sixth in China.^[2]^ Of all malignant pancreatic tumors, pancreatic ductal adenocarcinoma (PDA) that originates in the pancreatic ductal epithelium constitutes more than 90% and presents a high degree of malignancy and more than 50% of cases presents distant metastasis when initially diagnosed, leading to an unfavorable death within 1 year.^[3]^ The 5-year survival rate of patients with PC are less than 5% if without any treatment.^[4]^ While optimal surgical resection could improve 5-year survival rate up to 25% to 35% after surgery which has been considered to provide the only chance of long-term survival.^[5]^ However, although patients with PDA had accepted curative surgical resection, the prognosis remains poor since the disease generally advances or recurs, resulting in only 10% to 20% of 5-year survival rate.^[6]^ Unfortunately, only 15% to 20% of diagnosed PC patients can be potentially curative resection since PC is often advanced when initially diagnosed.^[7]^ Although the treatment strategy of the disease has been remarkably improved, the treatment outcome is rather limited and prognosis of patients with PC remains extremely poor.

Chemokines play an important role in regulating wound healing and inflammation by recruiting neutrophils to inflammatory sites. Growth regulated oncogene (GRO) belongs the chemokine ligand family and there are 3 different kinds of subtypes (GRO-α, GRO-β, and GRO-γ, respectively) and now are also described as CXCL1, CXCL2, and CXCL3, accordingly. Among these proteins, all 3 ligands bind to the common receptor CXCR2, a G protein-coupled chemokine receptor, while GRO-α/CXCL1 has the highest affinity. GRO-α is a 73-amino acid 8-kDa protein which is structurally and functionally related to GRO-β/CXCL2, GRO-γ/CXCL3, and interleukin-8.^[8]^ Originally, the GRO-α protein was extracted and purified from supernatant of a cultured human melanoma cell line as a autocrine growth factor^[9]^ and was found to be an inflammatory factor that plays a critical role in wound healing by modulating cell migration and angiogenesis,^[10]^ GRO-α was also shown to be an oncogene in a Chinese hamster embryonic fibroblasts model by subtractive hybridization technology^[11]^ and has multiple effects on cell proliferation, tumor angiogenesis, invasion, and metastasis.^[12,13]^

GRO-α has been reported to be over-expressed in a series of human cancers including colorectal cancer,^[14]^ melanoma,^[15]^ breast cancer,^[16]^ bladder cancer,^[17]^ gastric cancer,^[18]^ hepatocellular carcinoma,^[19]^ and ovarian cancer.^[20]^ Furthermore, GRO-α is known to regulate multiple biologic activities associated with tumor progression including primary tumor growth, tumor angiogenesis, tumor cell proliferation, and development of metastatic disease, and some studies have suggested a role in tumor prognosis.^[18,19,21,22]^ To the best of our knowledge, GRO-α expression in pancreatic cancer along with its correlation with clinicopathologic characteristics have not been evaluated to date. In the current study, 24 fresh frozen-tissues (including 12 pancreatic cancer tissues and 12 normal control tissues) were prepared to examine the expression of *GRO-α* mRNA by quantitative real-time polymerase chain reaction (qRT-PCR). Additionally, 160 formalin-fixed, paraffin-embedded (FFPE) samples of pancreatic cancer tissue and 68 control specimens, including 37 matched normal surgical margins and 31 benign pancreatic lesions were prepared for tissue microarrays (TMA) construction and determined the expression of GRO-α protein using immunohistochemistry (IHC). Finally, we analyzed the relationship between GRO-α and clinical characteristics of patients with PC. Our findings suggest that the GRO-α protein level represents a novel indicator of poor prognosis and may be a potential target gene for cancer therapy in patients with PC.

## 2 Materials and methods

### 2.1 Collection of clinical information and preparation of tissue microarrays

All patients who were hospitalized with pancreatic cancer between January 2004 and May 2012 were enrolled by the Department of Pathology, Affiliated Hospital of Nantong University, Jiangsu, China. Each patient had undergone pancreatectomy and none of patients had treated with radio therapy or chemotherapy before operation. All resected specimens were diagnosed with pancreatic cancer and then determined the pathological tumor node metastasis (TNM) stage using the criteria of the seventh version of the Union for International Cancer Control (UICC) TNM Classification Of Malignant Tumors, 2009. All patients had been closely follow-up after surgical treatment until their death or the beginning of this article. Subsequently, we selected 12 fresh pancreatic cancer tissues and 12 surrounding normal tissues as controls for mRNA determination by qRT-PCR. Another 160 FFPE tissue samples of pancreatic cancer and 68 control specimens, including 37 matched normal surgical margins and 31 benign pancreatic lesions, were prepared as TMA for GRO-α protein detection by IHC method.

The current study was authorized by the Human Ethical Research Committee. Informed consent was in advance attained from all patients.

### 2.2 RNA extraction and qRT-PCR analyses

As previously described,^[23]^ 24 fresh frozen tissues (including 12 fresh pancreatic cancer and 12 surrounding normal tissues) were prepared for RNA extraction by RNeasy Plus Mini Kit (74134, Qiagen, Germany). Total RNA was subsequently reverse transcribed into complementary DNA (cDNA). 18S rRNA (4453320, Life Technologies) was purchased and applied as an internal standard. qRT-PCR was presented with the SYBR Green PCR Master Mix (Toyobo, Osaka, Japan) according to the standard protocol and amplified with target gene-specific primers. The primer sequences for GRO-α were 5′- GAT TGT GCC TAA TGT GTT -3′ (sense) and 5′- ATC CAG ATT GAA CTA ACT TG -3′ (antisense). The reaction cycles were as follows: 10 minutes at 95 °C for Taq activation, after that, 40 cycles were carried out with 15 seconds at 95 °C and 60 seconds at 60 °C. The whole process was done in triplicate.

### 2.3 Tissue microarray construction

As previously described,^[23]^ a total of 160 FFPE PC tissues and 68 matched normal tissues were used for TMA construction. The Tissue Microarray System (Quick-Ray, UT06, UNITMA, Korea) was performed to manufacture 2 mm thick, FFPE pancreatic cancer TMA sections. Then, the individual TMA sections were moved to the new slides and numbered in sequence, preparing for IHC staining.

### 2.4 Immunohistochemical staining and scoring

IHC staining was performed as previously described.^[23]^ Firstly, deparaffinage, antigen retrieval, and quenching the activity of endogenous peroxidase with 3% H_2_O_2_ for 20 minutes were carried through in turn for the TMA slides. Then the slides were incubated with the primary anti-GRO-α antibody (0.5 μg/mL, ab 86436, Abcam, Hong Kong) at 4 °C overnight. After washing with phosphate-buffered saline (PBS), horseradish peroxidase (HRP) labeled anti-rabbit secondary antibody was subsequently added at room temperature for 30 minutes. Finally, slides were colorized using 3,3-diaminobenzidine (DAB) and restained with hematoxylin. For negative controls, PBS was used instead of the primary antibody.

The results of IHC staining were evaluated under an optical microscope by 2 independent, trained pathologists in a double-blind method. Expression levels of GRO-α protein were analyzed as previously described.^[24]^ Briefly, in each individual TMA section, the percentage of GRO-α positive cell was scored as follows: 0 represents 0% staining, 1 represents 1% to 33%, 2 represents 34% to 66%, and 3 represents 67% to 100%. The intensity of GRO-α staining was also scored as follows: 0 represents no color, 1 represents mild staining, 2 represents moderate positive staining, and 3 represents intensely positive staining. Thus, we defined that the product of above 2 components as the final score for further data analysis.

As previously described,^[23]^ the X-tile software was performed to determine the cutoff value for GRO-α protein expression score which was statistically significantly correlated with overall survival (OS).

### 2.5 Statistical analyses

The SPSS20.0 software was presented to calculate for data statistical analysis. A paired t test was performed for comparison of GRO-α mRNA expression and a Pearson χ^2^ test was performed to differ from GRO-α expression between cancerous pancreatic tissues and normal pancreatic tissues, and for the correlation between GRO-α and clinicopathologic characteristics. Univariate and multivariate analyses were presented with the Cox proportional hazards regression model. The Kaplan–Meier method was performed to construct overall survival curves. In the current study, of all analyses, *P* < 0.05 was deemed to be statistically significant.

## 3 Results

### 3.1 Analysis of GRO-α mRNA expression in pancreatic cancer by qRT-PCR

**Figure 1:**
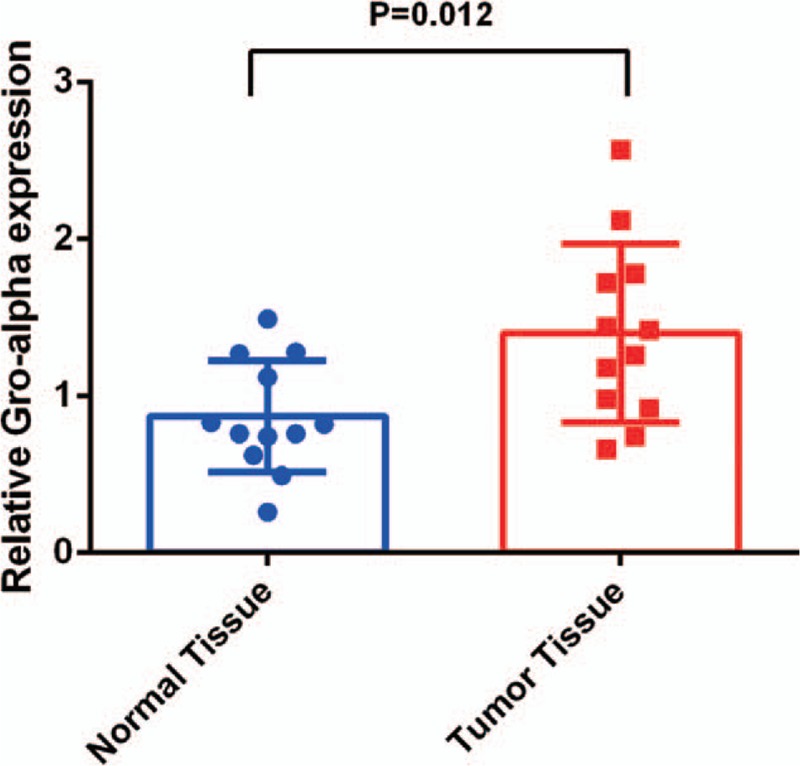
Mean *GRO-α* mRNA expression level normalized to that of 18S rRNA by qRT-PCR. GRO-α = growth-regulated oncogene-alpha, qRT-PCR = quantitative real-time polymerase chain reaction.

qRT-PCR was performed to detect the expression of *GRO-α* mRNA in pancreatic cancer and corresponding adjacent normal tissues. We observed that the expression of *GRO-α* mRNA relative to expression of the 18 seconds internal control rRNA was higher in pancreatic cancer tissues than that in adjacent normal tissues (1.399 ± 0.165 vs. 0.870 ± 0.103; *t* = 1.75, *P* = 0.012). The average level of *GRO-α* mRNA was 1.61-fold higher in pancreatic cancer compared with tumor-adjacent normal tissues (Fig. 1).

### 3.2 Detection of GRO-α expression in pancreatic cancer by IHC

**Figure 2:**
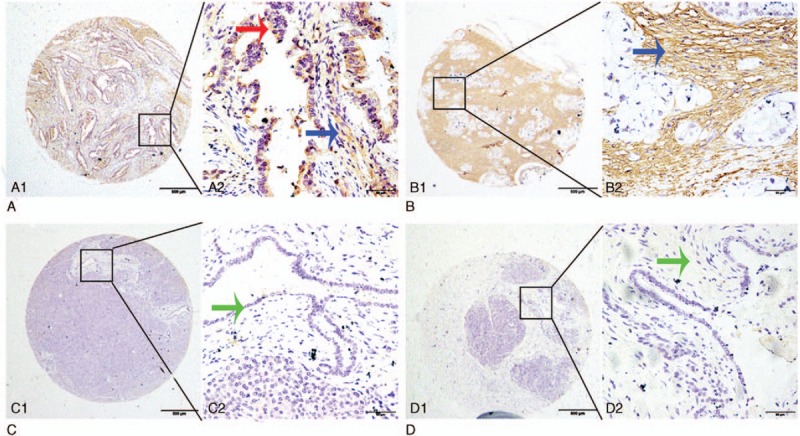
Representative pattern of GRO-α expression in pancreatic cancer (PC), adjacent noncancerous tissue, and benign pancreatic disease tissues by immunostaining of TMA sections. A1 and A2, Strong positive tumor cytoplasmic immunohistochemical staining (red arrow) and stromal staining (blue arrow) of GRO-α in PC samples. B1 and B2, Strong stromal immunohistochemical staining (blue arrow) of GRO-α in PC samples. C1 and C2, Negative staining for GRO-α in epithelial cells (green arrow) in adjacent noncancerous tissue. D1 and D2, Negative immunohistochemical staining for GRO-α in benign pancreatic stromal cells (green arrow). Original magnification ×40 (bar = 500 μm) in A1, B1, C1, and D1; ×400 (bar = 50 μm) in A2, B2, C2, and D2. GRO-α = growth-regulated oncogene-alpha.

To investigate the expression levels and location of GRO-α protein in cancer tissues, IHC was performed on TMA paraffin-fixed of 160 pancreatic cancer tissues and 68 matched non-cancerous specimens. GRO-α protein was primarily expressed in the cytoplasm of cancer cells and stromal cells, presented as brown particles, while low or no positive signals were detected in the nuclei of cancer cells and no positive signals in normal pancreatic ductal epithelial cells (Fig. 2).

Here, we defined 60% as the cutoff point in GRO-α level in cytoplasm of cancer cells and in stroma (*P* = 0.008 and *P* = 0.002, respectively) by using X-tile software program. Finally, we defined that the score from percentage and intensity which was higher than 60% as high expression, on the contrary, the score which was lower than 60% was considered as low expression. High GRO-α expression in the cytoplasm of cancer cells was detected in 41.88% (67/160, Table 1) of cancerous samples, compared with 8.8% (6/68, Table 1) of matched control samples, and high expression of GRO-α in the stroma was detected in 40.62% (65/160, Table 1) of cancerous samples compared with 25.00% (17/68, Table 1) of normal control samples.

**Table 1:**
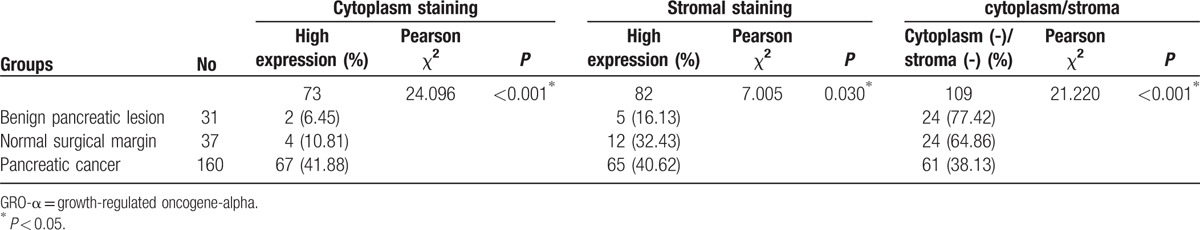
Cytoplasmic and stromal staining of GRO-α expression in pancreatic benign and cancerous tissues.

### 3.3 GRO-α protein expression in pancreatic cancer

Although high GRO-α protein expression was detected in benign pancreatic lesions and normal surgical margins, the frequency of high GRO-α protein expression in the cytoplasm of cancer cells and stroma was significantly higher in pancreatic cancers (*P* < 0.001 and *P* = 0.030, respectively; Table 1).

### 3.4 Relationship between GRO-α protein expression and clinical characteristics

**Figure 3:**
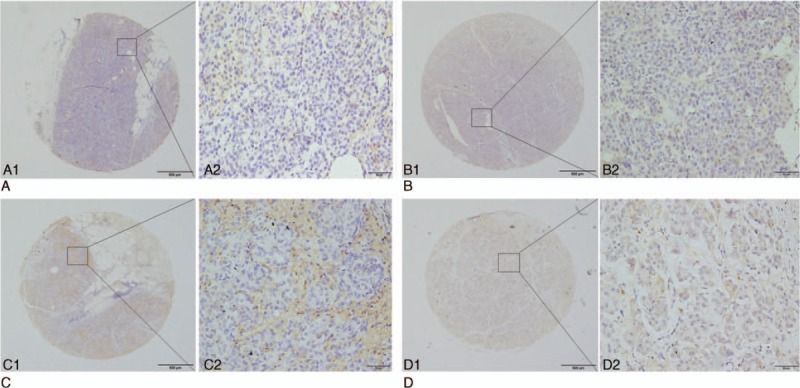
GRO-α expression in pancreatic cancer (PC) with different TNM stages by IHC. A1 and A2 (PC patient with TNM stage I A) showed light positive expression of GRO-α, and B1 and B2 (TNM stage II A) showed mild to moderate positive expression of GRO-α, while C1 and C2 (TNM stage III) and D1 and D2 (TNM stage IV) showed strong positive expression of GRO-α. Original magnification ×40 (bar = 500 μm) in A1, B1, C1, and D1; ×400 (bar = 50 μm) in A2, B2, C2, and D2. GRO-α = growth-regulated oncogene-alpha, IHC = immunohistochemistry, TNM = tumor node metastasis.

The relationship between levels of GRO-α protein and the clinicopathologic parameters of pancreatic cancer is shown in Table 2. Pearson χ^2^ analysis showed that positive GRO-α expression in the cytoplasm of cancer cells was inversely correlated with tumor location (*P* = 0.047), but positively correlated with T classification (*P* = 0.001), distant metastasis (*P* < 0.001), and TNM stage (*P* < 0.001, Fig. 3). In contrast, no significant correlation was found for sex, age, degree of differentiation, perineural invasion, vascular invasion, and lymphatic metastasis (Table 2). High GRO-α expression in the stroma was correlated with perineural invasion (*P* = 0.010), T classification (*P* = 0.006) and TNM stage (*P* = 0.004, Fig. 3), and was marginally associated with distant metastasis (*P* = 0.056), while there was no significant correlation with sex, age, tumor location, degree of differentiation, vascular invasion, or lymphatic metastasis (Table 2).

**Table 2:**
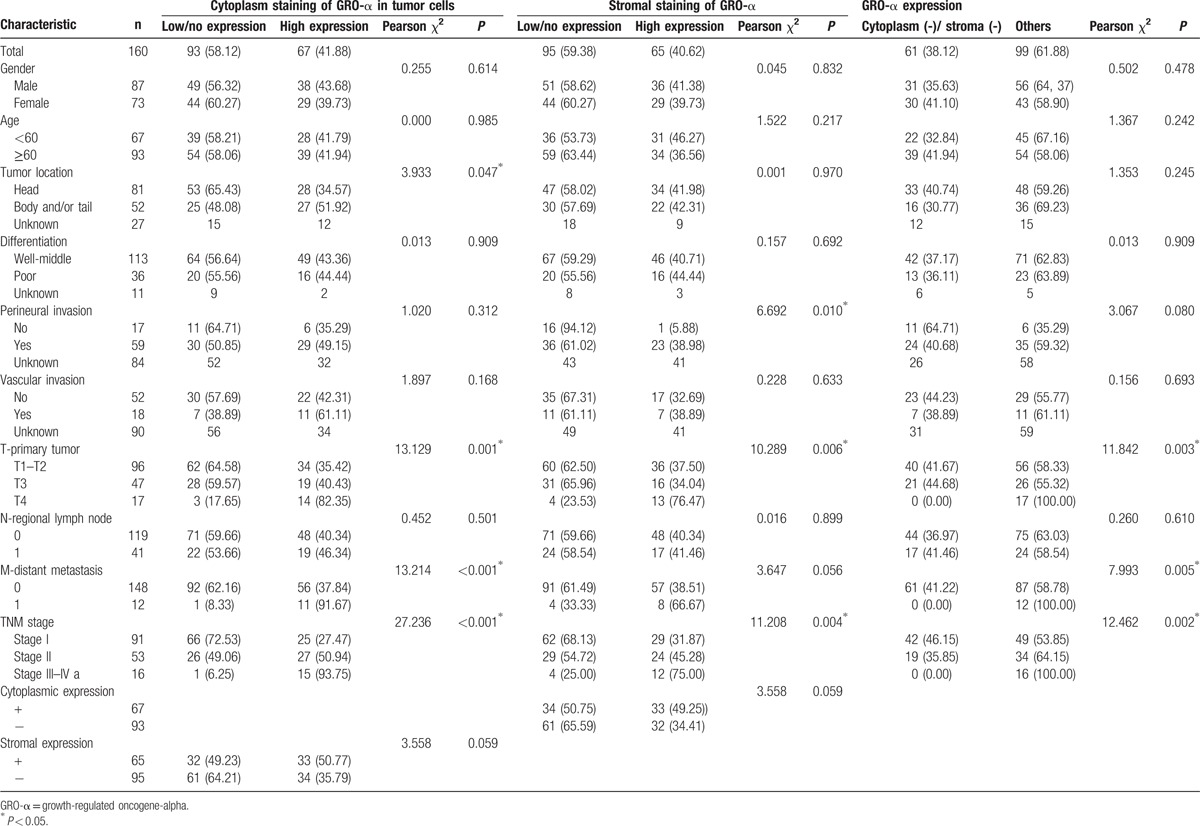
Relationship between the expression of GRO-α and clinicopathological characteristics in pancreatic cancer.

Low or absent GRO-α expression in both the cytoplasm of cancer cells and stroma (61/160 patients) was associated with T classification (*P* = 0.003), distant metastasis (*P* = 0.005), and TNM stage (*P* = 0.002; Table 2).

### 3.5 Over-expression of GRO-α in pancreatic cancer is associated with poor prognosis

Univariate analysis showed a correlation between T classification (*P* = 0.010), lymph node metastasis (*P* = 0.030), TNM classification (*P* = 0.040), low or absent GRO-α co-expression (*P* = 0.012), and positive GRO-α expression in the cytoplasm of cancer cells (*P* = 0.008) or stroma (*P* = 0.002) and the lifespan of patients with pancreatic cancer (Table 3). Multivariate Cox regression analysis further demonstrated that high GRO-α expression in the cytoplasm of cancer cells (HR = 5.730, *P* = 0.007) or stroma (HR = 3.120, *P* = 0.022), T classification (HR = 2.130, *P* = 0.023), lymphatic metastasis (HR = 4.211, *P* = 0.009), and TNM classification (HR = 0.481, *P* = 0.031) were independent prognostic factors for overall survival (Table 3).

**Table 3:**
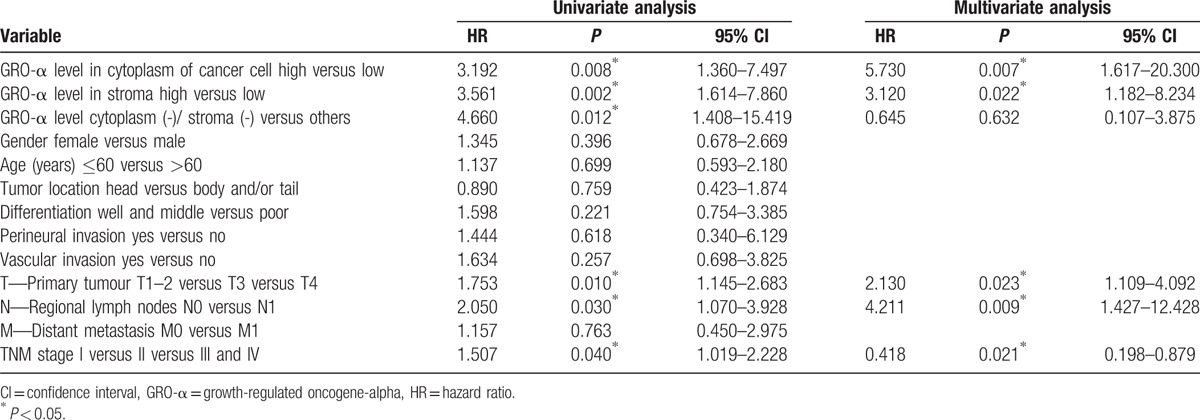
Univariate and multivariate analysis of prognostic markers for overall survival in pancreatic cancer patients.

**Figure 4:**
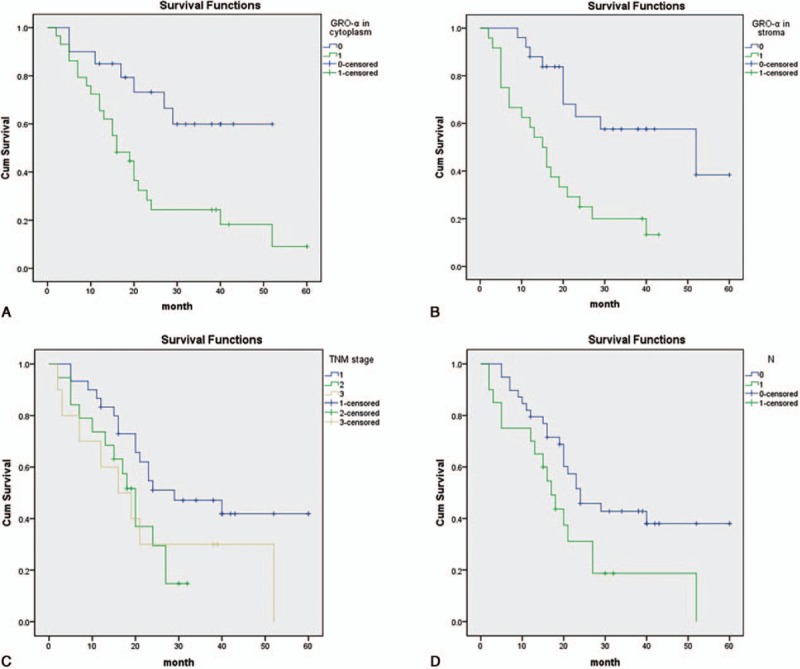
Survival analysis of PC patients by the Kaplan–Meier method and log-rank test. (A) Overall survival rate in patients with high cytoplasmic GRO-α expression (green line) was significantly lower than that in patients with low or no cytoplasmic GRO-α expression (blue line). (B) Overall survival rate in patients with high stromal GRO-α expression (green line) was significantly lower than that in patients with low or no stromal GRO-α expression (blue line). (C) Overall survival rate in patients with advanced stage of TNM (Stage II [green line] and Stage III–IV [yellow line]) was significantly lower than that of patients with early stage (Stage I, blue line). (D) Overall survival rate in patients with node metastasis (green line) was significantly lower than that in patients with no node metastasis (blue line). GRO-α = growth-regulated oncogene-alpha, TNM = tumor node metastasis.

Kaplan–Meier survival curves showed that pancreatic cancer patients with positive GRO-α expression in the cytoplasm of cancer cells exhibited significantly poorer survival time than those with negative GRO-α expression (Fig. 4a), and that high levels of GRO-α in the stroma were also associated with an unfavorable survival time (Fig. 4b). In addition, the OS rate in patients with advanced stage of TNM (Stage II [green line] and Stage III–IV [yellow line]) was significantly lower than that of patients with early-stage disease (Stage I, [blue line]) (Fig. 4c). Moreover, patients who underwent node metastasis (green line) had a significantly poorer survival time than those with no node infiltration (blue line; Fig. 4d).

## 4 Discussion

To date, potential predictive molecular markers for pancreatic cancer have been extensively investigated and some are identified to be significantly correlated with clinicopathologic characteristics and survival,^[25]^ but there is little information on the prognostic value of GRO-α.

Members of GRO protein family are detected in many cancer types, and GRO-α is frequently over-expressed in many cancers, including squamous cell carcinoma,^[13]^ colorectal cancer,^[14]^ breast cancer,^[16]^ prostate cancer,^[17]^ and ovarian cancer.^[20]^ In addition, GRO-α is involved in many biologic activities. For example, GRO-α is involved in carcinogenesis of melanoma.^[15]^ In epithelial ovarian cancer, GRO-α activates the mitogen-activated protein kinase signaling via trans-activating the epidermal growth factor receptor (EGFR), thus leading to cancer cell proliferation.^[26]^ In gastric cancer, GRO-α reinforces expression of vascular endothelial growth factor (VEGF) via activating the JAK2-STAT3 signaling and ultimately stimulates angiogenesis and tumor growth.^[27]^ Additionally, this chemokine was found to act as an effective mediator of tumor-associated angiogenesis in Kaposi's sarcoma^[10]^ and colorectal,^[28]^ breast^[29]^, and non-small-cell lung cancers (NSCLC).^[30]^ In a previous study,^[31]^ researchers detected that levels of CXCL1, 2, 3, 5, and 8 were elevated in tumor cells and high expression of CXCL1 tend to inhibit cell viability, invasion, and proliferation via down-regulating the most markedly up-regulated group member conduced by ShRNA. And down-regulation of CXCL1 led to strongly preventing from tumor growth in vivo. Altogether, these evidences confirm that CXCL1/GRO-α plays an important role in various malignant tumors through its involvement in tumor generation, proliferation, migration, and invasion. On the basis of these findings, we suggest that GRO-α expression might also act as a considerable accelerator in pancreatic cancer. In the current study, firstly the qRT-PCR was performed in small samples of cancerous and benign pancreatic tissues to examine the expression of *GRO-α* mRNA and showed a markedly increased level in cancerous tissues. Further, we generated tissue microarrays from resected pancreatic cancer specimens and normal control samples for IHC staining. Consistent with the qRT-PCR result, higher GRO-α protein expression was found in pancreatic cancer than in adjacent noncancerous tissues, indicating that GRO-α promoted development of pancreatic cancer.

Furthermore, we investigated the correlation between GRO-α expression and OS in patients with pancreatic cancer. Multivariate analysis showed that positive GRO-α expression in cytoplasm of cancer cells, positive GRO-α expression in stroma, T classification, lymphatic metastasis, and TNM classification were markedly correlated with the OS of patients with pancreatic cancer. Kaplan–Meier analysis indicated that the OS of PC patients with positive GRO-α expression was evidently shorter compared with those of negative expression.

CXCR2, the receptor of CXCL1, CXCL2, and IL8, has primarily been studied in leukocytes, including neutrophils,^[32]^ monocytes,^[33]^ and macrophages,^[34]^ in association with inflammatory diseases and immune responses. However, up-regulation of CXCR2 has also been correlated with tumorigenesis, cancer tissue angiogenesis, and metastasis of several cancers, including prostate,^[35,36]^ ovarian,^[20]^ and pancreatic^[37]^ cancers. Over-expression of CXCR2 also predicted a poor OS and disease-free survival (DFS) in patients with high-grade serous ovarian cancer.^[20]^ In gastric cancer,^[38]^ researchers reported that patients with increased expression of GRO-α together with its receptor CXCR2 was significantly correlated with tumor progression and with more advanced TNM stages, and concordantly, patients with lower GRO-α and CXCR2 expressions had a relative better prognosis. Consistent with this, high expression of GRO-α in breast cancer is always related to an unfavorable survival.^[39]^ In a study of patients with NSCLC,^[40]^ patients with malignant pleural effusion (MPE) had a short median survival time and increased regulatory T cells (Treg). The authors showed that the miR141-CXCL1-CXCR2 pathway regulates progression of NSCLC, and that decreased level of miR141 correlates with the survival of NSCLC patients with MPE, resulted in increased expression of CXCL1 and recruitment of Tregs to facilitate immune escape of the tumor.

Nonetheless, the contribution of GRO-α and its receptor CXCR2 to clinical features and survival of patients with pancreatic cancer remains obscure, and further studies are required for precise evaluation of the therapeutic and prognostic values of GRO-α and CXCR2 in pancreatic carcinoma.

In conclusion, to our knowledge, the current study is firstly to evaluate GRO-α expression in pancreatic cancer. Our findings indicated high expression of GRO-α in pancreatic cancer tissues, which was associated with a poor prognosis for patients. GRO-α may be a novel therapeutic target and has potential as a valuable prognostic biomarker of pancreatic cancer. Further research is necessary to clarify the precise mechanisms of action of GRO-α in pancreatic cancer.
